# Multibraided Fixed Retainers with Different Diameters after Magnetic Resonance Imaging (MRI): In Vitro Study Investigating Temperature Changes and Bonding Efficacy

**DOI:** 10.3390/dj12080255

**Published:** 2024-08-13

**Authors:** Maria Francesca Sfondrini, Maurizio Pascadopoli, Paola Gandini, Lorenzo Preda, Domenico Sfondrini, Karin Bertino, Cinzia Rizzi, Andrea Scribante

**Affiliations:** 1 Unit of Orthodontics and Pediatric Dentistry, Section of Dentistry, Department of Clinical, Surgical, Diagnostic and Pediatric Sciences, University of Pavia, 27100 Pavia, Italy; francesca.sfondrini@unipv.it (M.F.S.); paola.gandini@unipv.it (P.G.); karin.bertino01@universitadipavia.it (K.B.); cinzia.rizzi01@universitadipavia.it (C.R.); 2 Diagnostic Imaging and Radiotherapy Unit, Department of Clinical, Surgical, Diagnostic and Pediatric Sciences, University of Pavia, 27100 Pavia, Italy; lorenzo.preda@unipv.it; 3 Department of Radiology, Fondazione IRCCS Policlinico San Matteo, University of Pavia, 27100 Pavia, Italy; 4 National Center for Oncological Hadrontherapy (CNAO), 27100 Pavia, Italy; 5 Maxillo-Facial Surgery Unit, Fondazione IRCCS Policlinico San Matteo, 27100 Pavia, Italy; d.sfondrini@smatteo.pv.it; 6 Unit of Dental Hygiene, Section of Dentistry, Department of Clinical, Surgical, Diagnostic and Pediatric Sciences, University of Pavia, 27100 Pavia, Italy

**Keywords:** orthodontic multibraided retainer, magnetic resonance imaging, in vitro study, shear bond strength, adhesive remnant index

## Abstract

Objectives: Orthodontists are often asked to remove fixed retainers before patients undergo magnetic resonance imaging (MRI). The present in vitro study was designed to analyze the heating and bonding efficacy of stainless steel multibraided fixed retainers after 1.5- and 3-tesla (T) MRI. Materials and methods: A total of 180 human mandibular incisors were used to create 45 specimens of four teeth each, divided into nine groups. Handmade multibraided fixed retainers of three different sizes, defined by the diameter of the initial wire used (0.008″, 0.010″ and 0.012″), were tested. Three groups underwent MRI at 1.5 T, another three groups underwent MRI at 3 T and the last three groups did not undergo MRI. Temperature was assessed before and after MRI. Shear bond strength (SBS) and adhesive remnant index (ARI) were assessed after MRI for all groups. Data were statistically analyzed (*p* < 0.05). Results: After 1.5 T exposure, no significant temperature increase from T0 to T1 was observed in any of the groups (*p* > 0.05). Regarding the 3 T groups, a significant difference from T0 to T1 was found for all the groups (*p* < 0.05). Temperature changes were not clinically relevant, as they were less than 1 °C for all groups except for group 3 (ΔT0–T1: 1.18 ± 0.3 °C) and group 6 (ΔT0–T1: 1.12 ± 0.37 °C). Furthermore, there were no significant differences between the temperature variations associated with different wire diameters (*p* > 0.05). Conclusions: No significant changes in SBS or ARI were found (*p* > 0.05). Clinical significance: Since overheating was irrelevant and adhesion values did not change, the tested devices were concluded to be safe for MRI examinations at 1.5 T and 3 T.

## 1. Introduction

Magnetic resonance imaging (MRI) is a widely used diagnostic procedure that produces three-dimensional images without ionizing radiation to evaluate many medical conditions [1]. Orthodontic patients with bonded appliances are often asked to remove their devices prior to MRI examination [2]. As the stability of orthodontic treatment is an issue of great concern among clinicians, orthodontists usually employ fixed retainers to prevent relapse, as they seem to be the best choice to preserve long-term results [3,4]. As a matter of fact, in recent years, there has been an increase in the number of patients with fixed orthodontic retainers who, for various reasons, must undergo MRI examinations [5]. When objects are positioned in a magnetic field, they undergo a certain degree of magnetization that depends on their magnetic susceptibility [6]; in addition, metallic devices cause a loss of signal, resulting in a black spot on the image, called an artifact, which affects its diagnostic utility [7]. Furthermore, the interaction between metallic orthodontic appliances and the magnetic field of a scanner could cause their displacement, leading to injuries for the patients and potential damage to the radiographic device [8]. Finally, metals can cause heating [9]. Given that the majority of fixed orthodontic retainers are made of stainless steel [10] and that the effects of fixed retainers during MRI have not been fully investigated, orthodontists are frequently asked to remove them prior to MRI to avoid these risks [2]. On the other hand, the removal of these devices is associated with a number of problems, including damage to the enamel structure [11], wasted chairside time and money and unwanted tooth movement [9].

Currently, given the lack of guidelines in this field, orthodontists are committed to evaluating the effects of orthodontic devices in MRI in order to reduce the rate of removal [2,12]. The literature has evaluated artifacts from materials and devices used in orthodontics [5,6,13], but there is a paucity of data on fixed appliances, as the literature has focused on brackets and orthodontic bands [12,14,15,16]. Recent research suggests that the material of the retainer may have some influence on the generation of artifacts in head and neck MRI [17,18]. However, as no studies have been performed to evaluate the bond strength of stainless steel multibraided retainers subjected to MRI, the aim of the present study was to evaluate the in vitro effects of 1.5 T and 3 T MRI on the temperature and shear bond strength (SBS) of fixed retainers bonded to human teeth and the adhesive remnant index (ARI) values of these orthodontic appliances. The first null hypothesis was that there would be no significant difference in temperature before and after MRI at either 1.5 T or 3 T. The second null hypothesis was that there would be no significant difference in SBS scores between the groups before and after MRI. The third null hypothesis was that there would be no change in the frequency distribution of the adhesive remnant index (ARI).

## 2. Materials and Methods

The study was approved by the Unit Internal Review Board (2022-0223). A total of 180 permanent human lower incisors with intact enamel, no caries, no restorations and no fractures were collected and stored in 0.1% (*w*/*v*) thymol. The teeth were cleaned with a Softbrush Coarse 2140 (Edenta, Scarborough, ON, Canada) at low speed without water spray [19]. Four mandibular incisors were used as a base for the fabrication of fixed retainers, in which the teeth were embedded in marginal wax (Leone Spa, Sesto Fiorentino, Italy) and acrylic resin (Leocryl, Leone s.p.a.). This resulted in 45 samples. Fixed retainers were made by braiding 4 wires of preformed stainless steel ligature wires (Leone Spa) of different diameters: 0.008″, 0.010″ and 0.012″. Fixed retainers were placed on the vestibular surface of the incisors to facilitate the SBS test with a cutting force parallel to the fixed retainer. The vestibular enamel surface of each tooth was etched with 37% orthophosphoric acid gel (Gerhò Etchant Gel 37%, Gerhò Spa, Bolzano, Italy) for 30 s, followed by thorough rinsing with water for a further 30 s, as recommended by the manufacturer [20]. Transbond XT Light Cure Orthodontic Adhesive (3M Unitek, Monrovia, CA, USA) was then applied with a brush for 5 s and gently air-dried for 5 s, followed by polymerization with an LED light (Starlight Pro, Mectron SpA, Carasco, Italy) for 10 s. The retainer was then bonded with Transbond LR composite (3M Unitek), creating a circular area of 3 mm diameter and a total composite surface area of 7.065 mm^2^ (diameter/2 × π). Each area of composite was light-cured for 20 s [21]. The 45 specimens were divided into 9 groups (of 5 specimens each) according to the power of the MRI to which they were subjected and the dimension of the bonded retainer. The 9 groups are described in Table 1.

### 2.1. Temperature Test and MRI

All groups except groups 1, 4 and 7 (control groups) underwent MRI. Specimens were left in the magnet room for 24 h prior to MRI to normalize their temperature to the room. A contact thermometer (PeakTech^®^ Digital Thermometer 5135/5140 Prilf—und Messtechnik GmbH, Ahrensburg, Germany) was used to measure the temperature of each sample in degrees Celsius. The temperature of the samples was measured by placing the thermometer probe on the rail between elements 3.1 and 4.1. Temperature measurements were taken immediately before (T0) and after (T1) the MRI examination outside the magnet room. 

Samples from each group tested were placed in a plastic box inside the headrest of the patient in the machine. Groups 2, 5 and 8 underwent MRI at 1.5 T (model Magnetom Aera, Siemens AG, Munich, Germany), while groups 3, 6 and 9 underwent 3 T MRI (model Magnetom Skyra Fit, Siemens AG, Munich, Germany). The parameters used in the MRI are listed in Table 2 and Table 3. The total scan time was approximately 20 min for each group. After MRI and temperature measurement, all specimens were stored in saline to prevent dehydration of the enamel and to preserve its properties [22].

### 2.2. Shear Bond Strength (SBS) Test

**Figure 1:**
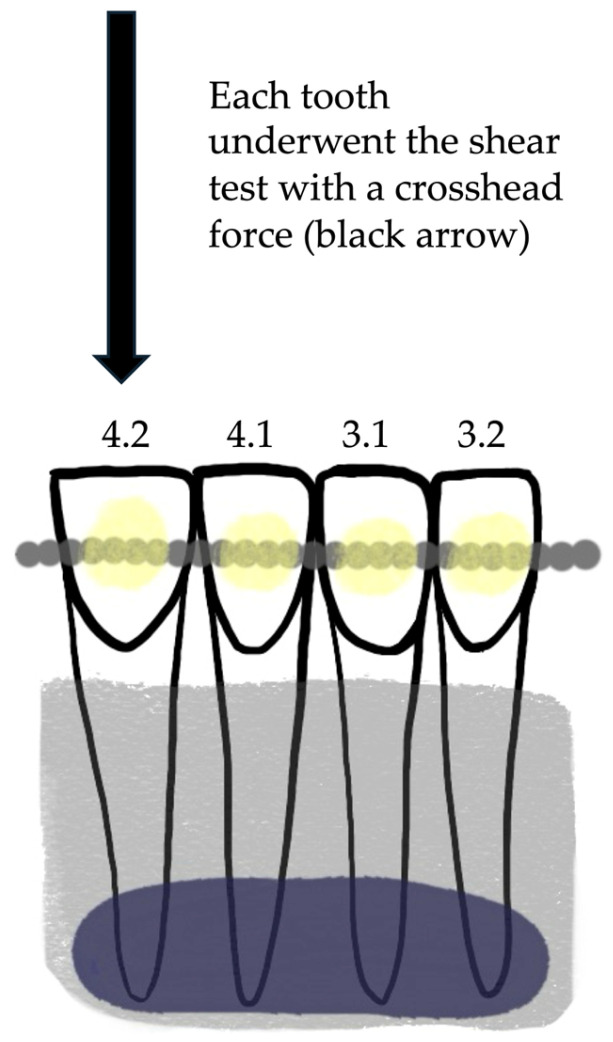
Example of one sample for the SBS test; teeth are numbered according to FDI World Dental Federation notation. Teeth were gathered with edging wax (violet) and acrylic wax (pale grey), while the retainers (grey) were bonded with composite (pale yellow) to the vestibular surfaces.

A universal testing machine (Instron Model 3343, Instron Corp, Canton, MA, USA) was used to perform the SBS test. Each specimen was fixed in the mechanical jaw with the fixed retainers parallel to the shear force. The maximum load required to debond the retainers was recorded in newtons (N) and converted into megapascals (MPa) using the known circular surface area of 7.065 mm^2^ [23]. The shear test was performed for each of the 45 samples first at the level of element 4.1, then at elements 4.2, 3.1 and finally 3.2. The same order was followed for all specimens. The crosshead speed was set at 1 mm/min [24,25]. Figure 1 shows the sample preparation and the SBS test.

### 2.3. ARI

After debonding, all specimens were examined by light microscopy (stereomicroscope SR, Zeiss, Oberkochen, Germany). The enamel and retainer were both evaluated and scored on a 0–3 scale [26]. This scale is used to define the interface by assigning each specimen a score of 0 (no adhesive left on the enamel and all adhesive left on the retainer), 1 (less than half of the adhesive left on the enamel and more than half of the adhesive left on the retainer), 2 (more than half of the adhesive left on the enamel and less than half of the adhesive left on the retainer), or 3 (all adhesive left on the enamel and no adhesive left on the retainer).

### 2.4. Statistical Analysis

Statistical analysis was performed using R software (version 3.1.3, R Development Core Team, R Foundation for Statistical Computing, Wien, Austria). Descriptive statistics, including mean, standard deviation, minimum, median and maximum values, were calculated for all groups. The normality of distributions was tested using the Kolmogorov–Smirnov test. Inferential statistics were performed using ANOVA and the Tukey post hoc test for temperatures and SBS values. Linear regression models were fitted for fixed retainer temperature and SBS, with wire diameter used and MRI power as covariates. The chi-squared test was performed to analyze ARI values. Significance was set at *p* < 0.05 for all tests.

## 3. Results

### 3.1. Temperature Test

**Figure 2:**
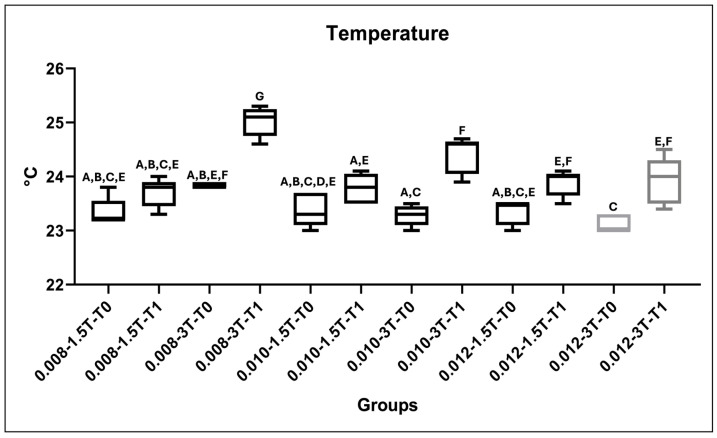
Graphical representation of temperature test for the tested groups and Tukey’s multiple comparison results. Plots with same capital letters do not show significant differences.

Descriptive statistics and the results of Tukey’s post hoc test on the recorded fixed retainer temperatures are shown in Table 4 and Figure 2. For the 1.5 T experiment, no significant increase from T0 to T1 was recorded for any of the groups (*p* > 0.05). For 3 T evaluations, a significant difference from T0 to T1 was found for all groups (*p* < 0.05). Temperature changes were not clinically relevant.

Table 5 shows temperature increases related to retainer diameter. There were no significant differences among temperature variations related to different wire diameters (*p* > 0.05).

Table 6, instead, shows a statistically significant temperature increase for 3T MRI in respect to 1.5T MRI (*p* < 0.05).

### 3.2. SBS Test

Descriptive statistics of SBS values are reported in Table 7. The Tukey post hoc test showed no significant difference in the SBS values among the various groups tested (*p* > 0.05).

### 3.3. ARI Test

**Figure 3:**
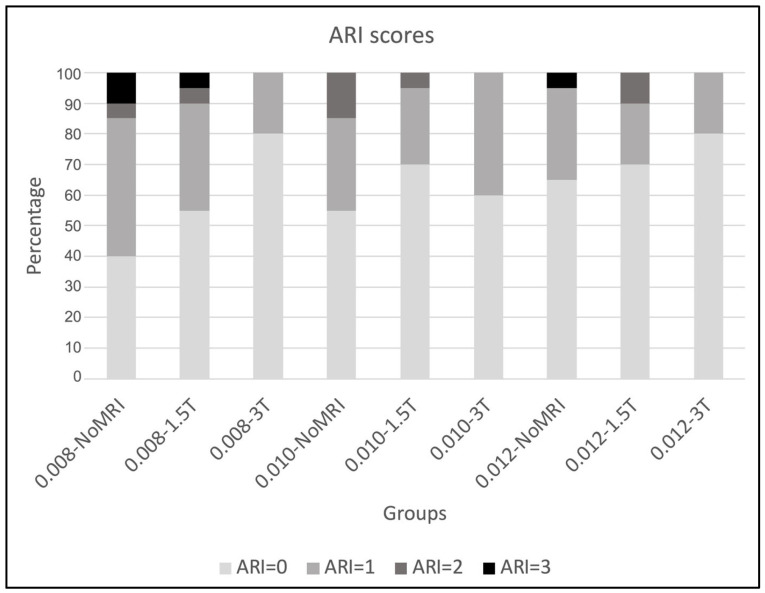
Bar plots of ARI scores.

The results of ARI scores are shown in Table 8 and Figure 3. The chi-square test revealed no significant differences among the scores (*p* > 0.05).

### 3.4. Linear Regressions 

**Figure 4:**
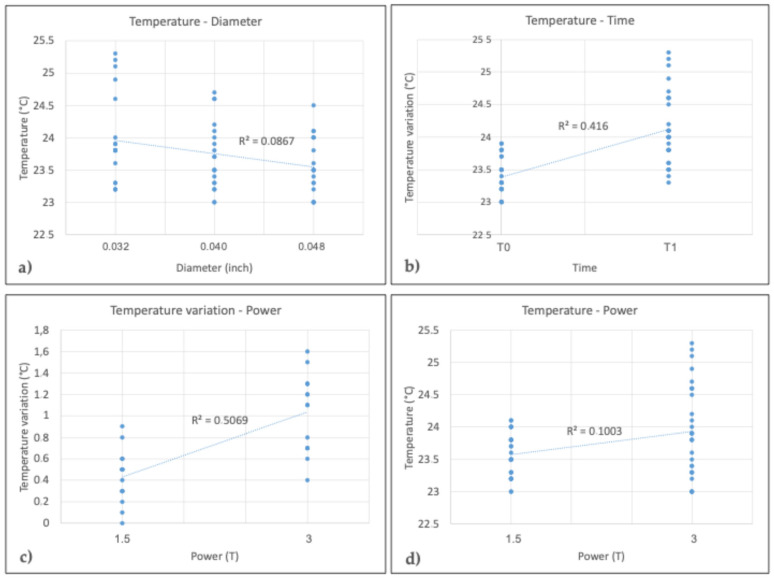
Plots of significant linear regressions: (**a**) temperature–diameter, (**b**) temperature–time, (**c**) temperature variation–power, (**d**) temperature–power.

Linear regression showed that temperature was significantly affected by the diameter of the retainer, the time and the power of the MRI scan (*p* < 0.05). Temperature variation, on the other hand, was only significantly affected by power (*p* < 0.05). Finally, SBS was not significantly affected by MRI power, retainer diameter or temperature variation (*p* > 0.05). Table 9 shows the exact *p* values of the linear regressions, while Figure 4 shows the plots of the regressions.

## 4. Discussion

Radiologists often require orthodontists to remove fixed orthodontic appliances, including brackets, molar bands and fixed retainers, even if they are distant from the anatomical area being examined, prior to performing an MRI because of the limited information in the literature regarding the impact of such materials on image quality and patient safety [2].

To date, research has focused on diagnostic imaging artifacts caused by materials and devices used in orthodontics, and it has been concluded in the literature that the presence of orthodontic appliances causes artifacts in MRI of the head and neck [27]. However, only a few studies have evaluated the potential for overheating and detachment of the devices themselves, mainly considering brackets and orthodontic bands [12,14,15,16].

To date, no author has investigated the changes in fixed retainer temperature and SBS after interaction with a magnetic field at 1.5 T and 3 T. Therefore, the purpose of the present in vitro study was to evaluate the thermal changes and SBS of stainless steel multibraided fixed retainers after 1.5 T and 3 T MRI. 

The first null hypothesis was rejected, as significant differences were found between the different retainers in terms of temperature increase. However, when the temperature changes of the retainer were examined, it was found that the temperature increase was always less than 1 °C for all samples, with the exception of group 3 (ΔT0–T1: 1.18 ± 0.3 °C) and group 6 (ΔT0–T1: 1.12 ± 0.37 °C).

These temperature changes are significantly lower than 5–7 °C, which would damage the dental pulp and cause necrosis [28,29]. Temperature changes were higher by a statistically significant margin at higher power levels (*p* < 0.05), but not to a clinically relevant degree. A possible explanation could be that an increase in magnetic field power corresponds to higher radiofrequency-induced heating [15]. 

On the other hand, the change in temperature as a function of splint diameter was not statistically significant (*p* > 0.05), but it should be noted that the average temperature difference decreased with increasing splint size. These results are partially consistent with the analysis performed by Sfondrini et al. in 2019, which showed that a 0.019″ × 0.025″ stainless steel archwire recorded the greatest increase in temperature, both when tested alone and when tested in combination with brackets. A 0.014″ stainless steel archwire, on the other hand, showed less overheating, suggesting that the temperature increased as the diameter of the arch increased [12]. The different results obtained in the present study may be due to a different interaction between the magnetic field and the braided wires used to make the brackets compared to the interaction with a single linear wire. 

However, the second null hypothesis was accepted, as no significant differences in SBS were found (*p* > 0.05). The lowest values of SBS were found in groups 3 (0.032″ and 3 T), 4 (0.040″ and no RM) and 9 (0.048″ and 3 T), showing that no significant influence of power on splint diameter was found. An exception to this finding was the 0.012″ retainer; its SBS values were found to decrease at 1.5 T and 3 T, with no minimum value for the 3 T examination, but no significant difference was found. Although information on the minimum clinically acceptable bond strength for retainers is limited [30], previous studies on brackets have shown that a bond strength of 6–8 MPa is sufficient to support orthodontic loads and intraoral stresses [31,32]. As the splints are subjected to continuous intraoral loading, the value found for orthodontic attachments/brackets can be considered the minimum SBS for retainers. In all the groups tested, the average values of adhesion force were higher than the accepted minimum values; therefore, if the adhesion between the enamel and the retainer is correct and is checked before the diagnostic examination, the attractive forces of the magnetic field should not affect the stability of the appliance.

As this is the first study to investigate this issue, it is not possible to directly compare the results obtained here with those of similar studies, but they can be related to a previous study [12], in which the minimum mean values related to SBS after MRI were above 6–9 MPa for each experimental test. The third null hypothesis was also accepted, as there was no difference in the ARI scores of the 9 groups. This finding seems to be directly related to the SBS scores, and thus, no specific behavior of the fixed retainers was found in this study, even though different dimensions were provided. It should be noted that the adhesion of the retainer to the vestibular surface may have been associated with a higher, although non-significantly higher, proportion of ARI 0 scores, despite the lack of significantly different SBS scores between the groups. Other authors have evaluated the safety of patients wearing orthodontic retainers during MRI, focusing on rotational and translational forces [33,34]. Specifically, in the 1.5 T and 3 T analyses, the forces generated are much greater than gravity, but adhesion plays a key role by acting as a counterforce, reducing the potential risk of retainer dislocation [33,34]. Therefore, the authors concluded that it is only necessary to check the adhesion and stability of the device before a patient undergoes MRI, without any obligation to remove the splint.

Radiologists must decide whether or not to remove an orthodontic appliance from a patient requiring MRI. Therefore, the aim of this report is to increase knowledge of the interaction between orthodontic appliances and MRI in order to assist radiologists and orthodontists in their work. There is only one guideline in the literature for the use of fixed orthodontic appliances in MRI from an official medical society [35]. This guideline states that for fixed orthodontic appliances, clinicians must first check that all parts are well fixed. Then, they must consider the materials of these appliances: ceramics and fiberglass do not interfere with the MRI examination, while metals may cause artifacts and heating, but they do not damage the dental pulp and are not attracted by the magnet during the examination. In terms of artifacts, fixed orthodontic appliances must be removed if they do not allow a correct assessment of the brain or maxillofacial area under investigation. In addition, current evidence suggests that titanium and gold retainers do not produce artifacts [17,18]; therefore, the material of the retainer should be carefully considered when patients are asked to undergo head and neck MRI. In the present study, only stainless steel retainers were evaluated, as they are still considered the standard for retention therapy [18]; further research should evaluate the clinical performance and bond strength of retainers made of other materials under MRI. Dobai et al. conducted a systematic review of the MRI compatibility of orthodontic brackets and wires. They concluded that heating of fixed orthodontic brackets and wires during 3 T or 1.5 T MRI is not harmful to orthodontic patients. They also concluded that debonding effects are not relevant [36].

In the present study, fixed retention appliances did not show clinically significant heating, adhesive forces did not change after MRI and appliances were not dislodged. 

Therefore, stainless steel fixed orthodontic retainers can be considered safe during MRI in terms of temperature increase and bond strength; clinicians could consider not removing them prior to 1.5 T and 3 T body MRI, except for head and neck imaging where there may be a risk of artifacts [2,17,18,37]. Notwithstanding, the current study has some limitations, the first being that it was designed as an in vitro study; therefore clinical trials are needed to confirm the findings reported here in terms of clinical failure. Another limitation is that the present study did not analyze artifact formation. Future studies should include the evaluation of artifacts according to different wire materials [17]. Moreover, the parameters adopted herein and the scanning sequence characteristics, which, at the moment, are not uniform with the current literature [17,38,39], make the present findings valid only under these conditions. Additionally, analyzing the interactions between orthodontic appliances and higher magnetic fields, such as those of the 6 T and T MRI machines that are now available and used in medical examinations, would be desirable [40].

## 5. Conclusions

The present experimental study showed statistically significant thermal changes with increasing MRI, but the changes were not clinically relevant. In fact, all temperature increases were moderate (0.36–1.18 °C) and therefore not risky for the patient in terms of pulpal viability and surrounding soft tissue; furthermore, the differences between SBS and ARI values, as measured after MRI, were not statistically significant and allowed sufficient adhesion of the fixed retainers during the procedure. 

## Figures and Tables

**Table 1:** Groups tested in the study.

Group	Wire Diameter (“)	MRI Exposure
1	0.032	Control (No MRI)
2	0.032	1.5 T
3	0.032	3 T
4	0.040	Control (No MRI)
5	0.040	1.5 T
6	0.040	3 T
7	0.048	Control (No MRI)
8	0.048	1.5 T
9	0.048	3 T

**Table 2:** Parameters of 1.5 T MRI.

1.5 T Parameters	T2-TSE Transverse	T1-TSE Transverse	T2-TSE Coronal	T1-TSE Transverse_ warp	T2-TSE Transverse_ warp	T2-FL 2D Hemo Transverse	Ep2d diff 7b-Value Transverse	T2-FLAIR Transverse	T1-VIBE 3D FS Transverse
FOV (mm)	240	240	180	240	240	210	340	240	240
Voxel size (mm)	0.5 × 0.5 × 3.0	0.5 × 0.5 × 3.0	0.6 × 0.6 × 2	0.5 × 0.5 × 3.0	0.5 × 0.5 × 3.0	0.5 × 0.5 × 3.0	1.1 × 1.1 × 4.0	0.5 × 0.5 × 3.0	0.6 × 0.6 × 0.6
Slice thickness	3.0	3.0	2.0	3.0	3.0	3.0	4.0	3.0	0.6
Slices	60	60	48	60	60	45	30	20	/
TE (ms)	108	8.6	79	8.6	110	25	81	94	2.46
TR (ms)	5640	739.0	8830	739.0	6280	1440	9700	4860	5.35
Scan time (min:s)	03:36	02:16	02:49	02:16	04:00	06:29	09:03	07:27	04:32

Abbreviations: T2 turbo spin echo in transverse projection (T2-TSE Transverse); T1 turbo spin echo in transverse projection (T1-TSE Transverse); T2 turbo spin echo in coronal projection (T2-TSE Coronal); warp, method for metal artifact reduction; T2 weighted two-dimensional fast low-angle shot for hemosiderin detection in axial projection (T2W-FL 2D Hemo Axial); echo-planar imaging two-dimensional with diffusion in transverse projection (Ep2d diff 7b-Value); (T2 fluid-attenuated inversion recovery in transverse projection (T2-FLAIR TRA); two-dimensional echo-planar imaging with 7 mm slice diffusion in transverse projection (EP2D DIFF 7 mm TRA); T1 volumetric interpolated breath-hold examination in three dimensions, fat-saturated (T1 VIBE 3D FS); field of view (FOV), time of echo (TE), repetition time (TR), turbo inversion recovery (TIR), and specific absorption rate (SAR) of the whole body.

**Table 3:** Parameters of 3 T MRI.

3 T Parameters	T2-TSE Transverse	T1-TSE Axial	T1-TSE Coronal	T2-FL 2D Hemo Axial	Ep2d diff 7b-Value Transverse	T2-TSE Transverse_ warp	T1-TSE Transverse_ warp	T2-FLAIR Axial	T1-VIBE 3D FS Axial
FOV (mm)	240	240	180	240	230	240	240	240	240
Voxel size (mm)	0.5 × 0.5 × 3.0	0.3 × 0.3 × 3.0	0.6 × 0.6 × 2	0.5 × 0.5 × 3.0	1.1 × 1.1 × 4.0	0.8 × 0.8 × 3.0	0.5 × 0.5 × 3.0	0.5 × 0.5 × 3.0	0.6 × 0.6 × 0.6
Slice thickness	3.0	3.0	2.0	3.0	4.0	3.0	3.0	3.0	0.6
Slices	52	52	39	45	20	52	52	40	
TE (ms)	104	10	75	12	58	94	8.1	90	2.48
TR (ms)	6260	689	7630	801	3300	5550	541	8000	530
TIR (ms)	/	/	/	/	/	/	/	2368	
Scan time (min:s)	03:09	02:52	02:26	03:11	03:02	03:21	02:19	02:56	04:26

Abbreviations: T2 turbo spin echo in transverse projection (T2-TSE TRA); T1 turbo spin echo in axial (T1-TSE Axial) and in coronal projection (T1-TSE Coronal); warp, method for metal artifact reduction; T2 weighted two-dimensional fast low-angle shot for hemosiderin detection in axial projection (T2W-FL 2D Hemo Axial); echo-planar imaging two-dimensional with diffusion in transverse projection (Ep2d diff 7b-Value); T2 fluid-attenuated inversion recovery in axial projection (T2-FLAIR Axial); T1 volumetric interpolated breath-hold examination in three dimensions, fat-saturated in axial projection (T1 VIBE 3D FS Axial); field of view (FOV), time of echo (TE), repetition time (TR), turbo inversion recovery (TIR), and specific absorption rate (SAR) of the whole body.

**Table 4:** Descriptive statistics of retainer temperatures (°C) in the various groups tested (T0, before MRI; T1, after MRI). * denotes Tukey’s multiple comparisons: means with the same letters are not significantly different.

Diameter (“)	Power	Time	Mean	SD	Min	Mdn	Max	Significance *
0.032	1.5 T	T0	23.34	0.26	23.20	23.20	23.80	A,B,C,E
		T1	23.70	0.26	23.30	23.80	24.00	A,B,C,E
	3 T	T0	23.84	0.05	23.80	23.80	23.90	A,B,E,F
		T1	25.02	0.28	24.60	25.10	25.30	G
0.040	1.5 T	T0	23.38	0.31	23.00	23.30	23.70	A,B,C,D,E
		T1	23.78	0.28	23.50	23.80	24.10	A,E
	3 T	T0	23.28	0.19	23.00	23.30	23.50	A,C
		T1	24.40	0.34	23.90	24.60	24.70	F
0.048	1.5 T	T0	23.34	0.23	23.00	23.50	23.50	A,B,C,E
		T1	23.88	0.24	23.50	24.00	24.10	E,F
	3 T	T0	23.12	0.16	23.00	23.00	23.30	C
		T1	23.92	0.43	23.40	24.00	24.50	E,F

**Table 5:** Descriptive statistics of thermal increases (in °C) in relation to diameter. * denotes Tukey’s multiple comparisons; means with the same letters are not significantly different.

Diameter (“)	Mean	SD	Min	Mdn	Max	Significance *	*p* Value
0.032	0.77	0.53	0.00	0.75	1.50	A	
0.040	0.76	0.46	0.20	0.65	1.60	A	
0.048	0.67	0.30	0.30	0.55	1.20	A	0.96

Moreover, ANOVA showed a significant difference in thermal increases related to 1.5 T and 3 T MRI power (*p* < 0.05).

**Table 6:** Descriptive statistics of thermal increases (in °C) in relation to MRI power. * denotes Tukey’s multiple comparisons; means with the same letters are not significantly different.

Power	Mean	SD	Min	Mdn	Max	Significance *	*p* Value
1.5 T	0.433	0.244	0	0.50	0.90	A	
3 T	1.033	0.358	0.4	1.10	1.60	B	<0.0001

**Table 7:** Descriptive statistics of SBS (in MPa) tested at different magnetic field powers. * denotes Tukey’s multiple comparisons: means with the same letters are not significantly different.

Group	Diameter (“)	Power	Mean	SD	Min	Mdn	Max	Significance *
1	0.032	Control	16.01	6.47	5.92	13.39	29.17	A
2	0.032	1.5 T	18.36	6.83	6.94	18.42	28.08	A
3	0.032	3 T	15.89	7.37	5.62	15.56	27.86	A
4	0.040	Control	15.52	5.80	6.94	12.91	28.36	A
5	0.040	1.5 T	20.21	10.62	7.45	17.81	53.09	A
6	0.040	3 T	18.05	5.47	7.79	18.44	26.42	A
7	0.048	Control	19.77	6.73	8.95	20.15	33.55	A
8	0.048	1.5 T	17.53	5.32	7.92	17.73	26.56	A
9	0.048	3 T	15.04	5.85	4.74	15.99	24.10	A

**Table 8:** Frequency of distributions (%) of ARI scores: 0, no adhesive left on enamel surface; 1, less than half of the adhesive left on the enamel; 2, more than half of the adhesive left on the enamel; 3, all the adhesive left on the enamel.

Diameter (“)	Power	ARI = 0	ARI = 1	ARI = 2	ARI = 3
0.032	No MR	8	9	1	2
0.032	1.5 T	11	7	1	1
0.032	3 T	16	4	0	0
0.040	No MR	11	6	3	0
0.040	1.5 T	14	5	1	0
0.040	3 T	12	8	0	0
0.048	No MR	13	6	0	1
0.048	1.5 T	14	4	2	0
0.048	3 T	16	4	0	0

**Table 9:** Linear regressions of the variables of the study. *: *p* < 0.05.

Dependent Variable	Independent Variable	*p* Value
Temperature	Diameter	0.0224 *
	Time	<0.0001 *
	Power	0.0137 *
Temperature variation	Power	<0.0001 *
Shear bond strength	Power	0.497
	Diameter	0.653
	Temperature variation	0.417

## Data Availability

Data are available upon reasonable request to the corresponding authors.
